# A novel periodized high‐repetition giant‐set resistance and high‐intensity interval training effects on the metabolic and pro‐inflammatory parameters in long‐ and short‐term overweight men

**DOI:** 10.1002/ejsc.12038

**Published:** 2024-01-30

**Authors:** Mahmoud Nikseresht, Mehdi Nikseresht

**Affiliations:** ^1^ Department of Exercise Physiology Ilam Branch Islamic Azad University Ilam Iran; ^2^ Department of Exercise Physiology Mazandaran University Babolsar Iran

**Keywords:** C‐reactive protein, exercise training, IL‐6 trans‐signaling, overweight history, periodization

## Abstract

This study compared the capacity of high‐repetition giant‐set resistance training (HGRT) and high‐intensity interval training (HIIT) to decline C‐reactive protein (CRP), interleukin (IL)‐6, soluble IL‐6 receptor (SIL‐6R), IL‐18, and insulin resistance markers in long‐ (5–10 years) and short‐term (1–4 years) overweight men. Another purpose was to compare these markers between the two populations. Firstly, eligible participants were matched based on age, body mass index, and aerobic fitness and then divided into a long‐ (*n* = 37) or short‐term (*n* = 32) overweight population. After that, the participants in each category were randomly assigned to HGRT (2 giant sets of 4‐exercise × 2 circuits at 30%–50% of one‐repetition maximum with a novel periodization), HIIT (4‐set × 4‐min of running at 80%–90% of HRmax), and control (CON, non‐exercising) groups. The well‐balanced training programs were performed in three weekly sessions for 12 weeks. This study showed that individuals with a longer history of being overweight have elevated baseline concentrations of CRP, SIL‐6R, and IL‐18 (all, *p* ≤ 0.04, effect size [ES] ≥0.65). Both training programs led to a similar reduction in insulin and homeostasis model assessment of insulin resistance versus CON (all *p* ≤ 0.03, ES ≥ 0.49) regardless of the duration of being overweight, although SIL‐6R was significantly reduced after HIIT versus pretest (both, *p* ≤ 0.03, ES = 0.17). The two exercises caused a significant decline in IL‐18 in the long‐term population versus CON, while HIIT was the only program that decreased CRP (all, *p* ≤ 0.04, 0.21 ≤ ES ≤ 0.34). Thus, it seems that overweight history plays a crucial role in deteriorating some of the biomarkers, and the two exercises have greater potential to attenuate IL‐18. However, HIIT might have a greater anti‐inflammatory effect (as indicated by CRP and SIL‐6R) than HGRT.

## Key points

- Participants with a longer history of being overweight had significantly higher C‐reactive protein (CRP), soluble IL‐6 receptor (SIL‐6R), and interleukin (IL)‐18, although there was no significant difference for IL‐6 and insulin resistance markers.
- The 12 weeks of high‐repetition giant‐set resistance training (HGRT) and high‐intensity interval training (HIIT) had equal effect on insulin resistance markers and obesity indices regardless of the duration of being overweight.
- The HGRT and HIIT led to a reduction of IL‐18 in the long‐term overweight population.
- HIIT was the only program that declined CRP and SIL‐6R.

## 1 INTRODUCTION

Obesity is one of the major risk factors to increase chronic metabolic diseases such as type 2 diabetes (T2D). Obese people can secrete more pro‐inflammatory cytokines, which leads to a damaged immune response and greater susceptibility to diseases than people with normal body weight (de Oliveira Dos Santos et al., 2021; Nikseresht, 2018). It has been shown that exercise training has a protective role against many diseases by reducing the concentration of these cytokines (Pedersen, 2017), although there are no exact mechanisms by which exercise training induces beneficial effects.

It has been accepted that regular exercise training can gradually decrease the concentration of pro‐inflammatory cytokines, especially in people with high levels at the beginning. For example, interleukin (IL)‐18 is an important pro‐inflammatory cytokine that activates NF‐kB and induces inflammatory properties (Kaplanski, 2018). There is also sufficient evidence for the function of this cytokine in the development of chronic diseases (Kaplanski, 2018). A cohort study recently showed that the concentration of IL‐18 was significantly higher in COVID‐19 patients, and it remains high for the next 3 months (Willems et al., 2022). Additionally, a study has shown a considerable decline in IL‐18 following 12 weeks of exercise training (40% of the program was endurance training [ET] and 60% was strength training [ST]) in subjects with metabolic syndrome (Trøseid et al., 2009). This cytokine, however, had a significant decrease after 3 months of high‐intensity interval training (HIIT) in obese people with the metabolic syndrome but it remained unchanged with resistance training (RT) (Stensvold et al., 2012). Therefore, the response of this marker may be different depending on the type of training.

IL‐6, a pleiotropic cytokine, has a key role in host defense because of its numerous immune functions (Reihmane & Dela, 2014). For example, several biological functions of this cytokine are regulated by its receptor system, which is classic or trans‐signaling (Reihmane & Dela, 2014). It attaches to its membrane‐bound receptor and exerts anti‐inflammatory properties through the activation of Janus kinase in the classic method. On the other side, it can also be linked to the soluble type of the IL‐6 receptor (SIL‐6R) that exerts pro‐inflammatory properties (Reihmane & Dela, 2014). A recent study illustrated that a very short‐term moderate‐intensity aerobic training program did not change IL‐6 (Mohamed & Alawna, 2021). Another study also showed no significant alterations in IL‐6 and C‐reactive protein (CRP) following 16 weeks of traditional endurance, strength, and concurrent (endurance plus strength) training in sedentary middle‐aged men (Libardi et al., 2012). Moreover, 12 weeks of nonlinear resistance and aerobic interval training did not change IL‐6 in obese men (Nikseresht et al., 2014). Reljic et al. (2022), however, reported that a 12 weeks of low‐volume HIIT program has higher efficacy for improving IL‐6 and CRP compared to single‐set RT, three‐set RT, and whole‐body electromyostimulation in individuals with the metabolic syndrome. In addition, these markers significantly decreased after 12 weeks of HIIT, RT, and a combination of both in T2D patients (Sabouri et al., 2021). On the other hand, data on the response of IL‐6R to exercise is very limited. For example, there was a significant decrease in SIL‐6R after 3 months of cycling training in heart failure patients (Adamopoulos et al., 2002), and after 2 weeks of HIIT in obese men (Leggate et al., 2012), but no study has examined the efficacy of RT on SIL‐6R. Although a relatively large number of studies have examined the inflammatory markers response to exercise, their results are contradictory. Therefore, recognizing accurate cutoff points among populations may tackle the issue. As a result, one of the hypotheses of the current study is that a longer history of being overweight can remarkably deteriorate inflammatory markers.

It is obvious that different kinds of training programs have different metabolic adaptations. These adaptations may differ depending on the exercise intensity, duration or volume, recovery between exercise intervals, and type of exercise. For example, exercise duration alone is responsible for a >50% of the fluctuation in IL‐6 after exercise (Fischer, 2006). It is important to claim that high‐repetition giant‐set resistance training (HGRT) allows higher repetitions with less recovery that emphasizes on muscular endurance, which might be a better choice for overweight people. In the present study, it is designed in such a way that its duration was the same as the HIIT. Despite obvious evidence for the positive adaptations of HIIT, the capacity of HGRT to reduce inflammatory markers has not been investigated. We hypothesized that the HGRT may improve the inflammatory markers as much effective as the HIIT. Thus, the main purpose of this study was to compare the effects of 3 months of HIIT and HGRT on serum CRP, IL‐6, IL‐6R, IL‐18, insulin, and glucose concentrations in long‐ and short‐term overweight men. On the other hand, to the best of our knowledge, there is no study that has investigated the impact of overweight history on inflammation. Therefore, the second aim was to compare these listed variables between men who are overweight in different durations.

## 2 METHODS

### 2.1 Design study

**FIGURE 1:**
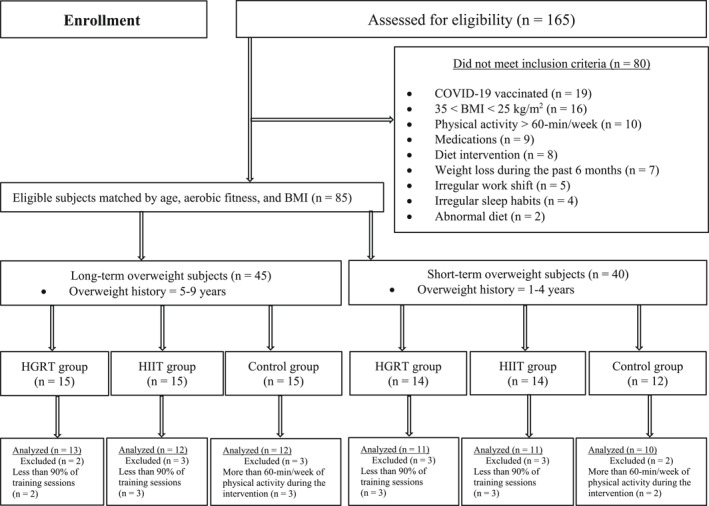
Flow diagram of this study. HGRT, high‐repetition giant‐set resistance training; HIIT, high‐intensity interval training.

The main purpose of this study was to investigate the effects of HGRT and HIIT on serum levels of selected cytokines, insulin resistance markers, aerobic capacity, and obesity indices in short‐term (1–4 years) and long‐term (5–9 years) overweight men. An additional aim was to compare these indicators between the different overweight populations. Firstly, volunteers were checked for the eligibility (Figure 1) and then divided into the two categories according to the overweight history. After being matched by age, body mass index (BMI), and aerobic fitness, the participants were allocated into short‐ and long‐term categories. After that, the participants were randomly assigned into one of the HGRT, HIIT, and control groups for each population. To investigate an exercise scenario, a period of 3 months of training protocols under controlled conditions was performed. These exercise programs were equalized for the duration of training. Participants in the control groups continued a sedentary lifestyle throughout the study. Blood samples were taken to detect serum levels of insulin, glucose, IL‐6, SIL‐6, IL‐18, and CRP at initial and post‐intervention points. To control confounding variables, all tests were accomplished by the researcher at the same time of the day.

### 2.2 Participants

Sixty‐nine eligible participants with different overweight durations analyzed in this study. The criteria for inclusion and exclusion are presented in Figure 1. To provide more information, they filled out a medical history and lifestyle questionnaire. For instance, only those who did not have a history of illness, smoking, especial diet (e.g., vegetarian) or regular exercise training during the past 6 months were selected as subjects. They also became familiar with all the procedures before any testing. The hazards for the research were clarified to them at the beginning. This study was confirmed by the local Research Ethics Council. All participants approved an informed consent depending on the human subject guidelines of our university.

### 2.3 Procedures

After fasting for a 10‐h period, body weight was measured with minimal clothes on a balance scale with an accuracy of <0.1 kg (Seca700 Mechanical Column Scale), and bare‐footed standing height was assessed using a wall‐mounted stadiometer with an accuracy of 0.1 cm. The distance between the last rib and iliac crest was assessed as abdominal circumference. To determine subcutaneous skinfold thickness, the chest, thigh, and abdomen areas were measured using a skinfold caliper in triplicate with a standard technique (Lange; Country Technology). The mean of the measurements was applied for each. Finally, the body fat percent (BF%) was calculated using Jackson and Pollock's formula (Jackson & Pollock, 2004). After a standardized warm‐up program, the VO_2_max was measured using a single‐stage treadmill‐walking test (Ebbeling et al., 1991). The warm‐up program was 10‐min jogging on a treadmill with some stretching exercises program. This test was re‐tested to rectify the effectiveness of fatigue and learning after a 4‐day period. The larger one was considered when there was a discrepancy.

Before and after the training, blood samples (10 mL) were obtained after an 8‐h sleep and a 10‐h fasting from the antecubital vein between 8 and 10 a.m. The participants lay in a supine position for a 15‐min rest before sampling. They were then questioned whether they consumed any medication or experienced any symptoms of illness for at least 5 days before the sampling. If a subject showed any problem during the period, it was postponed to 5 days later. Whole blood was centrifuged at 1000 rpm for 15 min (4°C), and the serum was taken and accumulated at −20°C until subsequent analysis. The values of biochemical variables were measured by the ELISA method in duplicate based on the manufacturer's guidelines. The R&D kit, Minneapolis, MN, USA, was applied to detect serum levels of IL‐6 and IL‐6R, while CRP and IL‐18 were detected with a conventional kit, Binding Site Group Ltd, UK, and Booster Biological Technology Ltd., Pleasanton, Calif., USA, respectively. The lowest diagnostic values were <0.08 ng/mL, 0.7 pg/mL, 1 pg/mL, and 0.01 mg/L for SIL‐6R, IL‐6, IL‐18, and CRP, respectively. To detect the concentration of insulin, a commercially assay kit (Q‐1, Diaplus) was applied. The inter‐ and intra‐assay coefficients of fluctuation was <5.1%. The concentration of glucose was assessed by the glucose oxidase procedure (Wako Pure Chemical). Finally, the homeostasis model assessment of insulin resistance was calculated [HOMA‐IR = insulin (mU/mL) × glucose (mmol/L)/22.5]. The samples were also taken at least 3 days after the last exercise session in the experimental groups (Gordon et al., 2013).

### 2.4 Dietary estimation

Only participants who had a normal diet were included in the study (Figure 1). Before the beginning of interventions, participants were instructed to consume a balanced dietary program (20%–30% fat, 50%–60% carbohydrate, and 10%–15% protein), which was analyzed by a software program (COMP‐EAT, National Analysis System, Version 4), and asked to sustain their daily calories throughout this study. They were also appealed to register their daily diet over a 7‐day period in the 5th and 10th weeks of the intervention to ensure a normal diet. Additionally, they were requested to eat an identical diet 2 days prior to the blood sampling (at baseline and after training).

### 2.5 Training programs

The participants in the well‐balanced HIIT and HGRT groups completed a 12‐week training program (Table 1), while the CON continued a sedentary lifestyle throughout the study. Individuals in the HIIT group performed four intervals of 4 min running on a treadmill at 80%–90% of maximal heart rate, with 3 min of recovery in between, which was monitored by using the Polar RCX5 sd Run, NY, USA. The HGRT program consisted of two circuits of two giant sets (four exercises for each) on major muscle groups at 30%–50% of one‐repetition maximum (1RM). Four main exercises (leg press, seated row, leg curl, and shoulder press) were performed in the first giant set without any rest in between that lasted 4 min, and it took roughly a minute for each exercise. After a 3‐min rest period, the second giant set (bench press, back extension, calf raises, and crunches exercises) was performed as the same as the first one. Then the next round was performed in the same way as the first circuit. Thus, the work‐to‐rest ratio in both training protocols was the same. The 1RM, which was assessed using the Brzycki method (Kraemer & Fleck, 2007), of the exercises was monthly reassessed and the resistance regulated accordingly. The participants were verbally persuaded to complete all the sessions. The training programs were held between 4 and 7 p.m. and monitored by the same researcher.

**TABLE 1:** Periodized high‐repetition giant‐set resistance training and high‐intensity interval training programs.

Training programs	Training variables	Weeks 1–4	Week 5	Weeks 6–8	Week 9	Weeks 10–12
HGRT	2 circuits × (giant sets^a^ 1 and 2)	2C × (giant sets 1 and 2 × 20R)	1C × (giant sets 1 and 2 × 20R) + 1C × (giant sets 1 × 20R)	2C × (giant sets 1 and 2 × 20R)	1C × (giant sets 1 and 2 × 20R) + 1C × (giant sets 1 × 20R)	2C × (giant sets 1 and 2 × 20R)
	• Exercises in giant set 1					
	Leg press, seated row, leg curl, and shoulder press, respectively					
	• Exercises in giant set 2					
	Bench press, back Extension, calf raises, and crunches, respectively					
	Intensity (% 1RM)	30	40	40	50	50
	Rest between giant sets (min)	3	3	3	3	3
	Duration (min)	28	20	28	20	28
HIIT	Volume (interval × min)	4 × 4	3 × 4	4 × 4	3 × 4	4 × 4
	Rest between intervals (min)	3	3	3	3	3
	Intensity (% MHR)	80	85	85	90	90
	Duration (min)	28	20	28	20	28

Abbreviations: 1RM, 1‐repetition maximum; C, circuit; HGRT, high‐repetition giant‐set resistance training; HIIT, high‐intensity interval training; MHR, maximal heart rate; R, repetition.

^a^ In each giant set, the four exercises were consecutively performed without any rest in between.

### 2.6 Statistical analysis

Normality of the distribution of data was checked using Shapiro–Wilk test. Independent‐sample *t*‐test was carried out to examine the discrepancy between the short‐ and long‐term overweight men. A one‐way ANOVA was applied to determine the difference between interventions at baseline. Furthermore, a two‐way repeated‐measures ANOVA was conducted to investigate the discrepancies between interventions and time points. When *F* ratios showed significant interactions (intervention × time) among means, a post‐hoc Bonferroni test was performed to determine where a difference occurred. The effect size (ES) was also measured for the interactions (intervention × time) to investigate the dignity of the intervention effects. To determine the within‐group reliability of the dependent variables at the two measurement stages, intraclass correlation coefficients (ICCs) were assessed. ICCs, for all variables, ranged between 0.51 and 0.98. All data analyses were performed using SPSS 25.0 (SPSS, Inc.). Furthermore, *p* < 0.05 was chosen as the significance level.

## 3 RESULTS

At baseline, all participants (*n* = 69, Table 2) were allocated in the long‐ (range = 5–9 years, *n* = 37) and short‐term (range = 1–4 years, *n* = 32) overweight populations. Serum levels of SIL‐6R, IL‐18, and CRP in long‐term participants was significantly higher than in short‐term participants (all, *p* ≤ 0.04), although there was no significant difference for VO_2_max, obesity indices, calorie intake, insulin resistance markers, and IL‐6 between the two populations (all, *p* ≥ 0.1).

**TABLE 2:** Comparison of the anthropometric, VO_2_max, and biochemical parameters between long‐term and short‐term overweight men at baseline.

Variables	Groups		Statistical		
	(Long‐term, *n* = 37)	(Short‐term, *n* = 32)	95% CI	*p*‐value	Effect size
Age (years)	28.4 ± 5.2	27.0 ± 5.9	−2.3 to 4.8	0.543	0.44
Overweight history (years)	7.3 ± 1.6^a^	2.9 ± 1.0	3.5 to 5.2	**0.0001**	3.21
Body weight (kg)	92.4 ± 6.3	90.1 ± 7.2	−3.6 to 5.9	0.112	0.96
Body mass index (kg/m^2^)	30.3 ± 5.1	28.7 ± 6.5	−3.5 to 7.2	0.155	0.91
Abdominal circumference (cm)	98.7 ± 7.1	97.3 ± 5.9	−5.7 to 8.0	0.123	0.59
Body fat (%)	28.5 ± 4.5	27.7 ± 4.6	−3.8 to 5.1	0.119	0.65
Energy intake (kcal/day)	2896 ± 287	2759 ± 212	−34.4 to 45.6	0.100	0.54
VO_2_max (mL/kg/min)	41.3 ± 5.2	42.4 ± 5.1	−3.4 to 2.8	0.114	0.22
Insulin (μU/mL)	6.7 ± 2.6	6.0 ± 2.6	−0.2 to 2.8	0.188	0.11
Glucose (mg/dL)	108.3 ± 16.6	101.8 ± 12.6	−5.9 to 15.0	0.339	1.13
HOMA‐IR	1.8 ± 1.2	1.5 ± 1.1	−0.4 to 1.0	0.387	0.26
IL‐6 (pg/mL)	2.7 ± 1.3	2.2 ± 1.9	−0.7 to 1.1	0.255	0.55
SIL‐6R (ng/mL)	51.6 ± 14.2^a^	43.5 ± 10.3	−0.5 to 16.8	**0.049**	0.65
IL‐18 (pg/mL)	98.5 ± 29.0^a^	65.2 ± 27.2	0.6 to 46.1	**0.022**	0.65
CRP (mg/L)	2.44 ± 0.61^a^	1.75 ± 0.44	0.01 to 0.69	**0.031**	0.66

*Note*: Data are presented as mean ± standard deviation. Bold values indicate less than 0.05 that is as a criteria.

Abbreviations: CI, confidence interval; CRP, C‐reactive protein; HOMA‐IR, homeostasis model assessment of insulin resistance; IL, interleukin; SIL‐6R, soluble interleukin‐6 receptor.

^a^ Significantly different between groups (*p* < 0.05).

Twelve weeks of HIIT and HGRT were equally effective at decreasing abdominal circumferences and BF% compared to CON in short‐ and long‐term individuals (all, *p* ≤ 0.03, Table 3). HIIT led to a significant decline in bodyweight and BMI compared to CON regardless of the duration of being overweight (all, *p* ≤ 0.04), while these variables remained unchanged with HGRT (both, *p* ≥ 0.1, Table 3). In the two populations, VO_2_max increased significantly after the training programs when compared to CON (all, *p* ≤ 0.02); however, this increase in HIIT was significantly higher than in HGRT (both, *p* ≤ 0.03, Table 3).

**TABLE 3:** Baseline and after training within‐ and between‐group comparison of the physical characteristics of short‐term and long‐term overweight subjects.

Variables	Overweight duration	Groups									*p*‐value			ES	SP
		HGRT			HIIT			Control			*t*	*i*	*t* ⨯ *i*		
		Mean ± *SD*	95% CI		Mean ± *SD*	95% CI		Mean ± *SD*	95% CI						
			Lower	Upper		Lower	Upper		Lower	Upper					
Body weight (kg)															
Baseline	Short‐term	87.1 ± 7.9	81.0	92.5	91.2 ± 8.8	85.2	96.7	92.8 ± 5.8	88.6	96.2	**0.001**	0.098	0.**001**	0.34	0.66
After training		87.8 ± 6.2	82.1	91.7	89.0 ± 7.4^a^ ^,^ ^b^	83.0	94.6	93.2 ± 6.4	87.1	97.6					
Baseline	Long‐term	88.8 ± 7.9	79.0	92.5	93.2 ± 7.8	85.1	99.7	94.1 ± 6.8	87.6	97.2	**0.001**	0.112	0.**001**	0.33	0.67
After training		87.8 ± 6.2	82.1	91.7	89.0 ± 7.4^a^ ^,^ ^b^	83.0	94.8	94.9 ± 6.7	87.1	97.6					
Body mass index (kg/m^2^)															
Baseline	Short‐term	28.2 ± 3.1	26.3	30.8	29.8 ± 3.4	26.0	31.6	28.7 ± 4.1	26.4	30.6	**0.03**	0.11	**0.04**	0.21	0.54
After training		28.7 ± 2.5	26.8	30.5	27.6 ± 2.1^a^ ^,^ ^b^	26.1	29.5	29.9 ± 4.4	27.5	31.2					
Baseline	Long‐term	29.2 ± 4.1	26.3	30.9	30.1 ± 4.4	26.7	31.9	29.1 ± 4.4	26.1	30.9	**0.02**	0.144	**0.04**	0.24	0.57
After training		28.7 ± 3.5	25.8	30.7	28.2 ± 2.1^a^ ^,^ ^b^	26.1	29.8	29.5 ± 4.9	27.1	31.6					
Abdominal circumference (cm)															
Baseline	Short‐term	96.1 ± 5.9	92.0	99.8	99.2 ± 6.7	94.6	104.1	98.2 ± 5.4	95.0	103.5	**0.001**	0.08	**0.001**	0.43	0.87
After training		93.8 ± 5.2^b^ ^,^ ^c^	90.8	97.2	94.4 ± 7.7^b^ ^,^ ^c^	90.0	98.8	98.7 ± 6.7	95.1	104.9					
Baseline	Long‐term	97.1 ± 5.7	92.7	99.7	99.7 ± 7.7	93.6	104.1	99.2 ± 5.4	93.0	103.1	**0.001**	0.11	**0.001**	0.55	0.87
After training		94.8 ± 5.7^b^ ^,^ ^c^	90.9	97.8	93.4 ± 8.7^b^ ^,^ ^c^	88.0	97.8	99.7 ± 6.7	92.1	104.9					
Body fat (%)															
Baseline	Short‐term	26.5 ± 4.3	23.6	29.2	28.0 ± 3.2	25.6	30.1	28.2 ± 2.6	26.0	30.5	**0.001**	0.09	**0.001**	0.47	0.97
After training		24.0 ± 4.0^b^ ^,^ ^c^	21.4	27.1	25.9 ± 2.9^b^ ^,^ ^c^	24.0	26.8	28.7 ± 3.0	26.1	30.9					
Baseline	Long‐term	27.9 ± 5.3	23.4	29.8	28.0 ± 3.2	25.6	30.1	29.2 ± 5.6	26.1	31.5	**0.001**	0.22	**0.001**	0.50	0.94
After training		24.0 ± 6.0^b^ ^,^ ^c^	22.4	27.7	25.9 ± 2.9^b^ ^,^ ^c^	24.0	26.8	29.7 ± 4.0	26.1	30.9					
VO_2_max (mL/kg/min)															
Baseline	Short‐term	42.7 ± 4.9	39.2	45.7	41.6 ± 6.3	37.2	46.0	43.6 ± 5.3	38.9	47.6	**0.001**	0.46	**0.001**	0.30	0.72
After training		45.1 ± 3.2^b^ ^,^ ^c^	43.4	47.3	46.7 ± 5.9^a^ ^,^ ^b^	42.1	50.3	43.1 ± 4.7	39.2	46.7					
Baseline	Long‐term	41.7 ± 5.9	37.2	45.1	40.6 ± 5.3	35.2	45.0	42.6 ± 6.3	36.9	47.1	**0.001**	0.68	**0.002**	0.37	0.77
After training		44.1 ± 4.2^b^ ^,^ ^c^	39.4	48.3	44.7 ± 4.9^a^ ^,^ ^b^	38.1	48.3	41.1 ± 6.7	37.2	46.9					

*Note*: Data are presented as mean ± SD. Bold values indicate less than 0.05 that is as a criteria.

Abbreviations: CI, confidence interval; ES, effect size; HGRT, high‐repetition giant‐set resistance training, HIIT, high‐intensity interval training; *i*, intervention; SP, statistical power, *t*, time.

^a^ Significantly different from other groups (*p* < 0.05).

^b^ Significantly different from baseline (*p* < 0.05).

^c^ Significantly different from the control group (*p* < 0.05).

**FIGURE 2:**
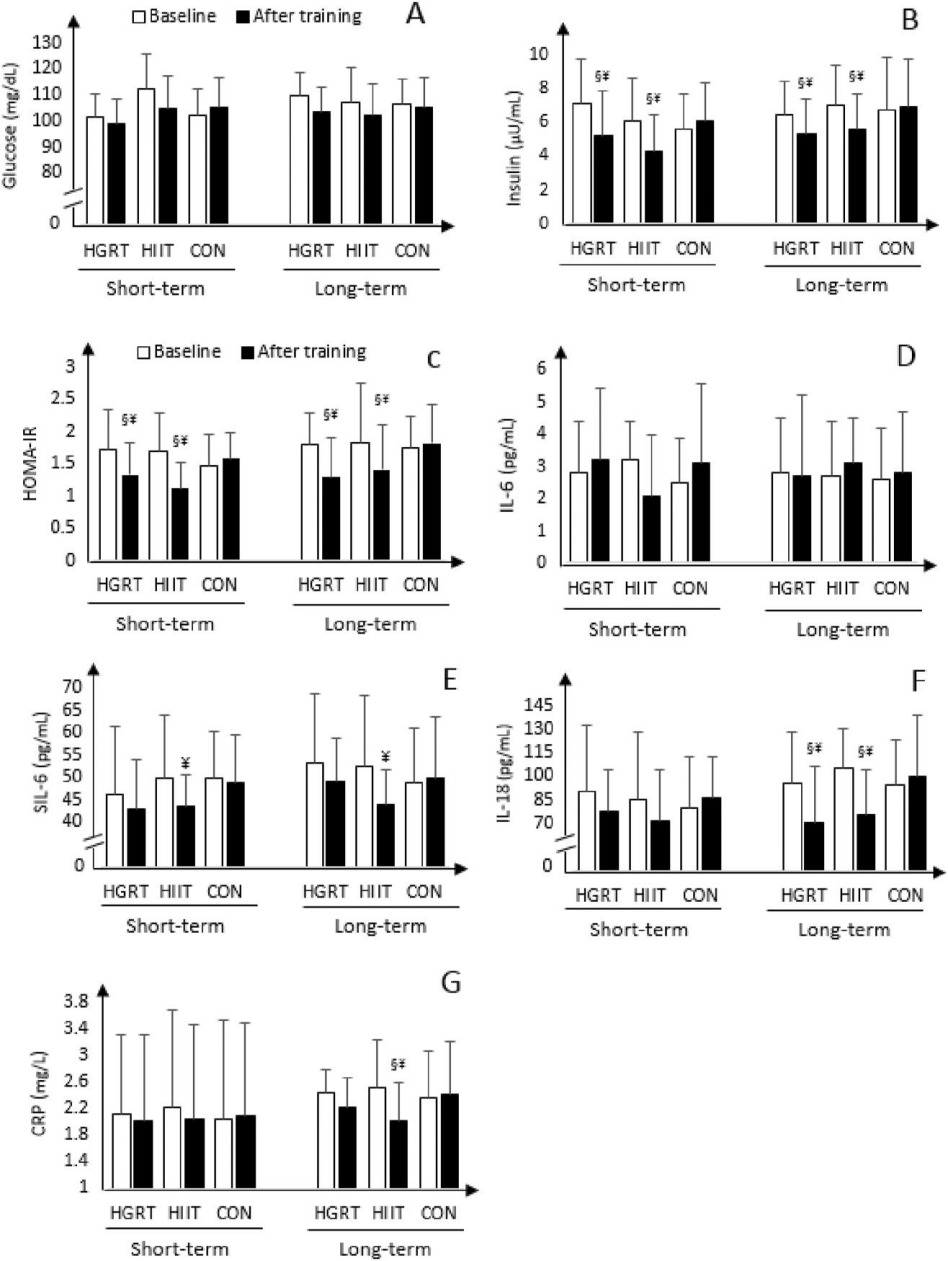
Baseline and after the training within‐ and between‐group comparison of the serum levels of glucose (A), insulin (B), HOMA‐IR (C), interleukin‐6 (D), SIL‐6R (E), interleukin‐18 (F), and CRP (G) in long‐term and short‐term overweight men. CRP, C‐reactive protein; HGRT, high‐repetition giant‐set resistance training; HIIT, high‐intensity interval training; IL, interleukin; SIL‐6R, soluble interleukin‐6 receptor. §: significantly different from the control group (*p* < 0.05). ¥: significantly different from baseline (*p* < 0.05).

There were improvements in insulin and HOMA‐IR in both HIIT and HGRT compared with CON regardless of the duration of being overweight (all, *p* ≤ 0.03, ES ≥ 0.49, Figure 2), while these training programs did not show any significant change for glucose and IL‐6 (all, *p* ≥ 0.3, ES ≤ 0.12, Figure 2). However, HIIT was the only protocol that had a positive impact on SIL‐6R in both short‐ and long‐term participants when compared to the baseline, albeit 4.3% more in long‐term overweight men (both, *p* ≤ 0.03, ES = 0.17, Figure 2). Compared to CON, IL‐18 was significantly declined after 12 weeks of HIIT and HGRT in long‐term participants (both, *p* ≤ 0.04, ES = 0.21, Figure 2), although HIIT was the only program that had a significant reduction in CRP (*p* = 0.03, ES = 0.34, Figure 2). However, there was no significant change in these inflammatory markers after the two exercises in short‐term participants (both, *p* ≥ 0.2, ES ≤ 0.27, Figure 2).

## 4 DISCUSSION

The study examined the capacity of 3 months of HGRT and HIIT to improve selected inflammatory cytokines and insulin resistance markers in short‐ and long‐term overweight men. The results indicated that both HGRT and HIIT were equally beneficial at decreasing abdominal circumference, BF%, insulin, and HOMA‐IR regardless of the duration of being overweight. A significant increase was found for VO_2_max after training programs, although the increase in HIIT was higher than that in HGRT in the two categories. Moreover, a significant reduction was observed in SIL‐6R after HIIT in comparison to the baseline in short‐ and long‐term populations. In long‐term participants, but not for the short‐term, the two exercise programs had a significant decline in IL‐18, although HIIT was the only program that had a positive effect on CRP. Another finding showed that men with a longer history of being overweight had significantly higher CRP, SIL‐6R, and IL‐18, while there was no significant difference for IL‐6 and insulin resistance markers between the two populations.

Three months of HGRT and HIIT did not significantly change in IL‐6 regardless of the duration of being overweight, which is in accordance with the study of Nikseresht et al. (2014). They showed that this cytokine remained unchanged after 12 weeks of nonlinear RT and HIIT in middle‐aged obese men. However, there was a significant decline in IL‐6 after 12 weeks of low‐volume HIIT in patients with the metabolic syndrome (Reljic et al., 2022). Likewise, this cytokine experienced a significant decrease following a 1‐year combined HIIT with RT and a moderate continuous training with RT in T2D (Magalhães et al., 2020). Similarly, IL‐6 significantly decreased after 3 months of HIIT (10 × 60‐s cycling intervals at the 85%–90% maximal heart rate), ST (seven main exercises at 75% 1RM), and combined training (firstly performed ST, followed by HIIT) in patients with T2D (Sabouri et al., 2021). In this study, there is no significant difference in IL‐6 between short‐ and long‐term populations, and the basal level of this cytokine is <3 pg/mL, which is much less than the quantities represented in other studies. Thus, these disagreements in findings might be induced due to differences in exercise programs or populations because factors including aging and diseases, especially T2D, can upregulate the cytokine. It is likely that a longer period of exercise training may have a higher capacity to reduce IL‐6, especially in subjects with a higher concentration at baseline.

In long‐term participants, IL‐18 significantly reduced after 12 weeks of HIIT and HGRT. Similarly, a study reported that a 16‐week aerobic training program (45–60 min sessions per week, 50%–85% VO_2_max) decreased this cytokine in overweight/obese patients with T2D (Kadoglou et al., 2007). In the same way, IL‐18 dropped following 12 weeks of exercise training (40% ET and 60% ST) in participants with the metabolic syndrome (Trøseid et al., 2009). On the contrary, 8 weeks of RT (four exercises of the major upper and lower body muscle groups, 30%–40% of 1RM) and ET (35 min of either ergometer cycling or treadmill walking at moderate intensity) programs, had no significant effect on this cytokine in patients with chronic obstructive pulmonary disease (Ryrsø et al., 2018). In addition, IL‐18 remained unchanged after 3 months of HIIT and nonlinear RT in men who were obese (Nikseresht et al., 2016). These discrepant results might be attributed to the variation in the initial value of IL‐18 and differences in populations. It is possible that a decreased IL‐18 response to exercise training is easier to achieve in participants with T2D or metabolic syndrome compared to sedentary obese people. This is because the value of this cytokine has increased in these patients. Moreover, in the present study, the initial level of IL‐18 in long‐term participants is higher than that in short‐term counterparts. Thus, it seems likely that regular exercise training is beneficial to reduce the cytokine in people with elevated levels to begin with.

On the other hand, CRP declined significantly only with HIIT in long‐term overweight individuals, which is in accordance with the study of Kohut et al. (2006). They reported that a 10‐month continuous aerobic training program, but not for RT, decreased CRP in elderly people. Two very recent studies also reported that 12 weeks of low‐volume HIIT and combined aerobic with RT had a significant decline in CRP in patients with the metabolic syndrome and T2D (Reljic et al., 2022; Su et al., 2022). However, there was no significant change in CRP after 9 weeks of HIIT (30 s sprint, 4–5 min passive recovery) or prolonged intermittent sprint training (10 s sprint, 2–3 min moderate exercise) in a middle‐aged, sedentary population (Allen et al., 2017). According to the data, it is recommended that initial levels of the biomarker should be considered because the basal value of CRP in long‐term men was higher than in short‐term men. The results of the present study indicate that HIIT may have much more potential to reduce IL‐18 in subjects with a higher concentration at baseline. Furthermore, the improvements of the VO_2_max and bodyweight/BMI after HIIT were higher than those in HGRT. A larger increase in VO_2_max or a lower increase in bodyweight or combination of both with HGRT as much as HIIT can be induced to equal effects. More studies are needed for better conclusions because this is the first study that examined the effect of overweight history on inflammation.

It has been recognized that IL‐6 can bind to SIL‐6R through the trans‐signaling pathway, which exerts pro‐inflammatory properties (Reihmane & Dela, 2014). We observed a comparable reduction in SIL‐6R only after HIIT when compared to the baseline in short‐ and long‐term overweight participants. This result suggests that the reduction of the SIL‐6R may be the reflection of the deactivation of the trans‐signaling pathway; as a result, another pathway is activated that has anti‐inflammatory effects. Thus, HIIT might have a higher beneficial effect on inflammation compared to HGRT. To support this, a similar decline was observed in SIL‐6R after a 3‐month cycling training program in stable heart failure patients (Adamopoulos et al., 2002). Moreover, it reduced after 2 weeks of HIIT in obese men (Leggate et al., 2012).

Insulin and HOMA‐IR significantly reduced after the training programs in short‐ and long‐term participants, but not for glucose. Similarly, 12 weeks of nonlinear RT and HIIT showed same results on these indicators in obese men (Nikseresht et al., 2014). Another study also revealed that 12 weeks of low‐volume HIIT protocol in 22‐min sessions at an intensity of 90% of VO_2_max was more effective than continuous aerobic training at an intensity of 60% VO_2_max in 36‐min sessions in HOMA‐IR in the metabolic syndrome (Gallo‐Villegas et al., 2018). On the other hand, glucose reduced significantly after 12 weeks of combined aerobic and RT in women with T2D (Su et al., 2022). It appears that the combination of aerobic and RT may be more effective at decreasing this marker compared to RT or aerobic training alone. Another explanation for the result is that the concentration of glucose is not very high in this study. Exercise programs may reduce the concentration of glucose in subjects with a higher level at the baseline. However, several responsible mechanisms, such as improving in post‐receptor insulin signaling, glucose transporter, messenger RNA synthesis, and the function of hexokinase and glycogen synthase have been already introduced (Dela et al., 1994). On the other hand, it seems that the training intensity is a key factor to reduce glucose because a high‐intensity exercise can easily increase glucose uptake from active muscle cells and consequently decline insulin resistance. To support this statement, a meta‐analysis study showed that HIIT is more effective at improving insulin resistance than continuous aerobic training, particularly in people who are at risk of T2D (Jelleyman et al., 2015). It is worth mentioning that, to gain a larger effect on insulin resistance, an increase in the exercise intensity is necessary. In the present study, the improvement of HOMA‐IR was accompanied by reductions in BF% and abdominal circumference in both interventions regardless of overweight history. Thus, this finding suggests that HIIT and HGRT can improve HOMA‐IR due to a reduction of the obesity indices.

Although there was no significant difference for IL‐6 and insulin resistance markers between short‐ and long‐term categories, long‐term men had a higher level of IL‐18, CRP, and SIL‐6R. Thus, it can be said that some cytokines react earlier to overweight history and some later. Therefore, this study showed for the first time that higher levels of these biomarkers in long‐term participants clearly show that the duration of being overweight can be considered as an important factor for inflammation. Another benefit of this randomized controlled study is to compare the efficacy of 3 months of HIIT and a new design of RT on selected metabolic parameters. On the other hand, this work is not without its drawbacks. For example, the number of samples in this research is relatively small, which might have led to the absence of efficacy on selected metabolic parameters. Another limitation is that the cutoff between the two populations (1–4 vs. 5–9 years) is a bit small. It is possible that a greater duration of being overweight may have more effects on these biochemical parameters. Hence, it is recommended that this issue be investigated further in future studies.

## 5 CONCLUSION

In summary, this study showed that participants with a longer history of being overweight had significantly higher CRP, SIL‐6R, and IL‐18, although there was no significant difference between IL‐6 and insulin resistance markers. In addition, 12 weeks of HIIT and HGRT, equalized in the training volume, had positive and similar effects on insulin resistance and obesity indices in the two categorized populations, while HIIT was the only program that led to a significant reduction in SIL‐6R. In long‐term men, however, both training programs could decrease IL‐18, but CRP declined significantly after HIIT only. This advantage is likely because of lower bodyweight/BMI or higher aerobic capacity with this training program. To conclude, this study could not support the idea that HGRT is as much effective as HIIT to improve these metabolic indicators. This study shows that a longer duration of being overweight may worsen the inflammatory status. However, both protocols had similar positive effects on most variables, but it seems that HIIT is a better strategy to attenuate some of them, especially in men with a longer history of being overweight.

## AUTHOR CONTRIBUTIONS

All authors met the conditions required for full authorship. **Mahmoud Nikseresht**: Methodology; validation; formal analysis; investigation; writing ‐ original draft; writing – review & editing; project administration. **Mehdi Nikseresht**: Investigation; validation; investigation; writing ‐ review & editing.

## CONFLICT OF INTEREST STATEMENT

The authors declare that no conflict of interest would prejudice their impartiality.

## Data Availability

Data and material are available on reasonable request to the corresponding author.
